# Soluble Fas might serve as a diagnostic tool for gastric adenocarcinoma

**DOI:** 10.1186/1471-2407-10-275

**Published:** 2010-06-10

**Authors:** Samaneh Boroumand-Noughabi, Hamid Reza Sima, Kamran Ghaffarzadehgan, Mostafa Jafarzadeh, Hamid Reza Raziee, Hanieh Hosseinnezhad, Omeed Moaven, Mohammad Taghi Rajabi-Mashhadi, Amir Abbas Azarian, Mojtaba Mashhadinejad, Jalil Tavakkol-Afshari

**Affiliations:** 1 Department of Pathology, Imam Reza Hospital, Mashhad University of Medical Sciences, Mashhad, Iran; 2 Gastric Cancer Research Group, Mashhad University of Medical Sciences, Mashhad, Iran; 3 Department of Internal Medicine, Imam Reza Hospital, Mashhad University of Medical Sciences, Mashhad, Iran; 4 Department of Pathology, Mashhad University Cancer Research Center, Omid Oncology Hospital, Mashhad University of Medical Sciences, Mashhad, Iran; 5 Young Researchers' Club, Medical School of Islamic Azad University of Mashhad, Mashhad, Iran; 6 Department of Radiation Oncology, Mashhad University Cancer Research Center, Omid Oncology Hospital, Mashhad University of Medical Sciences, Mashhad, Iran; 7 Department of surgery, Ghaem Hospital, Mashhad University of Medical Sciences, Mashhad, Iran; 8 Division of statistics, vice chancellery for research, Mashhad University of Medical Sciences, Mashhad, Iran; 9 Immunogenetic and Cell Culture Department, Immunology Research Center, Bu-Ali Research Institute, Mashhad University of Medical Sciences, Mashhad, Iran

## Abstract

**Background:**

Fas (Apo-1/CD95) and its specific ligand (FasL) are key elements in apoptosis. They have been studied in different malignancies but there are few published studies about the soluble forms of these markers (i.e. sFas/sFasL) in gastric cancer. We have compared the serum levels of sFas/sFasL in gastric adenocarcinoma patients and cases with pre-neoplastic lesions as potential markers for early diagnosis, and investigated their relation with clinicopathological characteristics.

**Methods:**

Fifty-nine newly-diagnosed cases of gastric adenocarcinoma who had undergone gastrectomy, along with 62 endoscopically- and histologically-confirmed non-cancer individuals were enrolled in this study. sFas/sFasL serum levels were detected by Enzyme Linked Immunosurbent Assay.

**Results:**

Mean serum sFas level was significantly higher in gastric cancer patients than in control group (305.97 ± 63.71 (pg/ml) vs. 92.98 ± 4.95 (pg/ml), P < 0.001); while the mean serum level of sFasL was lower in patients with gastric adenocarcinoma (0.138 ± 0.04 (pg/ml) vs. 0.150 ± 0.02 (pg/ml), P < 0.001). Mean serum levels of sFas/sFasL were significantly different in both intestinal/diffuse and cardiac/non-cardiac subtypes when compared to the control group (P < 0.001). There was an increase in the serum level of sFas from the first steps of pre-neoplastic lesions to gastric adenocarcinoma (P < 0.001). Patients who had no lymph node involvement (*N_0_*) showed significantly higher serum levels of sFas compared to others (P = 0.044).

**Conclusions:**

Production of sFas may play a critical role in the carcinogenesis of intestinal-type gastric cancer. sFas serum level may serve as a non-invasive tool for early diagnosis of gastric cancer.

## Background

Gastric cancer is the second leading cause of cancer-related mortality worldwide with a great geographic variation[1]. In Iran, it is the second most common cancer in males and the forth in females. It has been estimated that gastric cancer is the most common cause of cancer-associated mortality in Iranian population[2].

Although gastric cancer has a poor prognosis with a five-year survival rate of 25% in the United States[3], there is no standard biomarker for early diagnosis and no consensus on screening programs. To date, some guidelines have been proposed for early diagnosis[1] and new molecular markers and therapeutic strategies are required to design effective diagnostic and therapeutic protocols.

Derailment of apoptosis plays an important role in the development, growth and resistance of malignant tumors, and also influences the prognosis[4]. As a member of TNF-family receptors, Fas (Apo-1/CD95) is a cell surface protein that can induce apoptosis through its cytosolic tail after binding to its specific ligand, Fas Ligand[5]. Fas and Fas Ligand (FasL) are crucial in immune system homeostasis[6,7]. FasL is also a major weapon for cytolytic T cells to induce apoptosis in tumor cells[4]. Consequently, a decrease in Fas expression in the membrane of tumor cells can protect them from this lethal influence of FasL. FasL detection on the cell surface of some tumor cells proposed the hypothesis that these cells can escape immune attack through induction of apoptosis in tumor infiltrating lymphocytes[8,9]. The *Fas *gene produces two protein isoforms through alternative mRNA splicing: the full-length Fas which contains a trans-membrane domain, and the soluble form of Fas (sFas), which lacks this domain. Soluble Fas acts as a decoy in the extra-cellular environment and binds FasL[10,11]. Therefore, there is an immune privilege for tumor cells by secretion of sFas as an inhibitor of apoptosis.

Fas/FasL system has been investigated in a large variety of neoplasms[12-15]. However, few studies have been reported about gastric cancer to date. Previous studies indicate that gastric carcinomas express FasL at a higher level, while lower level of Fas expression leads to evade the killing effects of host immune system[16-18]. There are some conflicting reports about the correlations of Fas/FasL expression--studied by immunohistochemistry (IHC) method--and tumor size, depth of invasion, metastasis, differentiation and Lauren's classification of gastric tumors [18,19]. Few studies have been published about the serum level of sFas/sFasL in gastric cancer, with discrepancies in their results[20-23].

This study was conducted to assess the serum level of sFas/sFasL in gastric adenocarcinoma and non-tumoral lesions, to find their possible role in early diagnosis and their correlations with clinicopathological features of this malignancy.

## Methods

### Sample collection

Study included fifty-nine patients with newly-diagnosed, histologically-confirmed gastric adenocarcinoma, who were admitted to the department of surgery in Omid Oncology Hospital, Mashhad, Iran; between February 2006 and June 2008. Patients with unresectable tumors, history of previous chemotherapy, radiation therapy, or gastric surgery were excluded. Sixty-two individuals were enrolled in the control group. They had all undergone esophagogastrodeodenoscopy due to upper gastrointestinal complaints in the endoscopy unit, Imam Reza University Hospital, Mashhad, Iran; and were proven to have no endoscopic and histological evidence of gastric tumor.

The study protocol was approved by the Research Ethics Committee in Mashhad University of Medical Sciences. A written informed consent was obtained from each individual. Demographic characteristics (e.g. ethnicity, age, and gender) and clinical data including symptoms, medications, and potential risk factors were obtained via a questionnaire filled by trained personnel. These risk factors included history of tobacco consumption and family history of gastric cancer.

According to updated Sydney System[24], five biopsies were obtained from each individual in the control group for histological examination: two from the antrum, two from the corpus, and one from the incisura angularis. Another antral biopsy was obtained for the detection of *H. pylori *via a commercially available Rapid Urease Test (Chemenzyme Co., Iran). Biopsy samples were fixed in 10% buffered formalin. After routine tissue processing, they were examined for the presence of five pathologic variables including density of *H. pylori*, intensity of neutrophilic and mononuclear inflammation, atrophy, intestinal metaplasia, and dysplasia. We divided the control group into three subgroups according to the pathologic pattern: mild gastritis without *H. pylori *infection (named as near-normal mucosa), chronic gastritis with *H. pylori *infection (chronic active gastritis), and precancerous lesions (including chronic atrophic gastritis, gastric atrophy, intestinal metaplasia, and dysplasia). Giemsa staining was applied in suspicious cases for better evaluation of *H. pylori*.

In the cancer group, the histological diagnosis was based on morphological examination of the samples that were routinely processed and stained with hematoxylin and eosin method. According to Lauren's criteria, tumors were classified as intestinal and diffuse type. Tumor grade and surgical stage were determined as well.

### Enzyme Linked Immunosurbent Assay (ELISA)

A 3-ml sample of venous blood was collected from each participant before endoscopy or one day before surgery, in control and cancer groups, respectively. Immediately after blood sampling, serum was obtained by centrifugation at 2000 r/min for 15 min at 4°C and stored at -20°C until subsequent assay. The titres of *H. pylori *IgG antibody were measured via commercial ELISA (Padtan Elm Co., Ltd. Iran), according to the manufacturer's instructions. Serum levels of sFas and sFasL were assessed using human ELISA kits (Bender MedSystems, GmbH, Vienna, Austria) according to the manufacturer's instructions.

### Statistical analysis

Chi-square test with 95% confidence interval was performed for comparing the variables. As serum levels of sFas and sFasL did not have a normal distribution, we used Kruskal-Wallis and Mann-Whitney U tests to compare the mean serum levels of sFas and sFasL in different groups. Data have been shown as mean ± SEM (standard error of mean) in figures and tables. The statistical analyses were performed using the SPSS 16.0 statistical package (SPSS, Inc, Chicago, IL, USA).

## Results

Forty-four out of 59 patients with gastric adenocarcinoma were male (M/F ratio: 2.93). The median age was 62 years (ranging from 39 to 79, mean: 60.25 ± 10). Of 62 individuals in the non-tumoral group, 32 were males and 30 were females with a median age of 47 years (ranging from 20 to 77, mean: 47.32 ± 16). Poor economic status, lower level of education and tobacco consumption were significantly more prevalent in cancer group (p < 0.05). Most of our patients were diagnosed as intestinal type gastric cancer (45/59), of which 10, 15 and 5 were well, moderately and poorly differentiated carcinomas, respectively, and in 5 cases, the grade of differentiation was unknown. Among 62 non-cancer individuals 11 were categorized as near-normal mucosa, 35 as chronic active gastritis and in 16 cases, precancerous lesions were seen in the stomach. Based on laboratory exams, 73.6% of our cancer patients had a positive history of *H. pylori *infection, while in the control group this positive history was found in 82.3%.

Mean serum levels of sFas/sFasL based on descriptive characteristics of the non-tumoral and tumoral groups are summarized in table 1 and table 2. There was a positive correlation between tobacco smoking and mean serum level of sFas, but not sFasL, among non-tumoral group (P = 0.041). However, in the tumoral group, no association was observed between smoking and mean serum level of sFas/sFasL (P = 0.06 for sFas). There was no significant difference in serum levels of sFas/sFasL between cases with or without history of *H. pylori *infection in either tumoral or non-tumoral groups.

**Table 1:** Serum levels of sFas/sFasL based on descriptive characteristics of non-tumoral group

	Non-Tumoral Group					
	
	sFas *(pg/ml)*			sFasL *(pg/ml)*		
		
	N*	Mean ± SE.M^†^	P Value	N*	Mean ± SE.M^†^	P Value
**Gender**						
*Male*	30	103.21 ± 7.54	***0.023***	32	0.13 ± 0.02	0.09
*Female*	29	82.40 ± 5.88		30	0.17 ± 0.03	
**Economic Status\**						
*Poor*	15	85.21 ± 11.66	0.82	16	0.10 ± 0.03	0.17
*Moderate to Good*	43	95.07 ± 5.45		45	0.16 ± 0.02	
**Educational Status**						
*Under high school Diploma*	41	97.94 ± 6.44	0.25	43	0.16 ± 0.03	0.91
*High school Diploma & above*	16	81.42 ± 6.91		17	0.13 ± 0.02	
**Tobacco consumption**						
*Negative*	51	88.83 ± 5.03	***0.041***	53	0.16 ± 0.02	0.11
*Positive*	8	119.46 ± 15.27		9	0.11 ± 0.01	
**Opium addiction**						
*Negative*	46	86.81 ± 5.20	***0.015***	47	0.16 ± 0.02	0.52
*Positive*	13	114.82 ± 11.31		15	0.12 ± 0.02	
**History of *H. pylori *infection^‡^**						
*Negative*	49	76.17 ± 9.76	0.12	51	0.14 ± 0.04	0.27
*Positive*	10	97.74 ± 5.58		11	0.15 ± 0.02	

* N: Number of samples

^† ^Mean ± SE.M: Mean Serum Range ± Standard Error of Mean

^‡ ^History of H. pylori infection: Based on laboratory data

**Table 2:** Serum levels of sFas/sFasL based on descriptive characteristics of patients with gastric adenocarcinoma

	Gastric Adenocarcinoma Group					
	
	sFas *(pg/ml)*			sFasL *(pg/ml)*		
		
	N*	Mean ± SE.M^†^	P Value	N*	Mean ± SE.M^†^	P Value
**Gender**						
*Male*	41	348.89 ± 85.04	0.55	44	0.16 ± 0.06	0.28
*Female*	15	188.63 ± 4.54		15	0.06 ± 0.04	
**Economic Status**						
*Poor*	24	222.94 ± 45.98	0.93	26	0.05 ± 0.02	***0.006***
*Moderate to Good*	20	279.68 ± 104.11		21	0.20 ± 0.11	
**Educational Status**						
*Under high school Diploma*	38	262.75 ± 61.03	0.33	41	0.08 ± 0.03	***0.018***
*High school Diploma & above*	4	129.93 ± 35.48		4	0.60 ± 0.54	
**Tobacco consumption**						
*Negative*	28	234.81 ± 76.62	0.06	29	0.07 ± 0.03	0.34
*Positive*	15	281.3 ± 65.87		17	0.22 ± 0.13	
**Opium addiction**						
*Negative*	46	282.55 ± 64.73	0.37	49	0.15 ± 0.05	0.47
*Positive*	10	413.67 ± 202.46		10	0.10 ± 0.06	
**History of *H. pylori *infection^‡^**						
*Negative*	34	390.46 ± 145.66	0.64	37	0.10 ± 0.04	0.13
*Positive*	15	182.09 ± 21.08		15	0.14 ± 0.07	

* N: Number of samples

^† ^Mean ± SE.M: Mean Serum Range ± Standard Error of Mean

^‡ ^History of H. pylori infection: Based on laboratory data

The mean serum level of sFas was significantly higher in gastric cancer patients than control group (P < 0.001), while the mean serum level of sFasL was lower in patients with gastric adenocarcinoma (P < 0.001). After grouping the patients by histological type (intestinal/diffuse) and tumor location (cardia/non-cardia), there were statistically significant differences in serum sFas/sFasL level in all of the subgroups versus non-tumoral group (Table 3).

**Table 3:** Comparison of serum levels of sFas and sFasL in different subgroups of gastric adenocarcinoma with non-tumoral group

	sFas (pg/ml)			sFasL (pg/ml)		
		
	N*	Mean ± SE.M^†^	P Value	N*	Mean ± SE.M^†^	P Value
**Gastric Cancer Group**			(vs. Non-Tumoral)			(vs. Non-Tumoral)
*Overall*	56	305.97 ± 63.71	***< 0.001***	59	0.138 ± 0.04	***< 0.001***
*Intestinal*	43	293.06 ± 73.88	***< 0.001***	45	0.147 ± 0.05	***< 0.001***
*Diffuse*	13	348.61 ± 129.43	***< 0.001***	14	0.109 ± 0.05	***< 0.001***
*Cardia*	18	242.72 ± 62.78	***0.001***	18	0.026 ± 0.01	***< 0.001***
*Non-cardia*	37	358.92 ± 99.61	***< 0.001***	40	0.206 ± 0.07	***< 0.001***
						
**Non-Tumoral Group**	59	92.98 ± 4.95		62	0.150 ± 0.02	

* N: Number of samples

^† ^Mean ± SE.M: Mean Serum Range ± Standard Error of Mean

**Figure 1:**
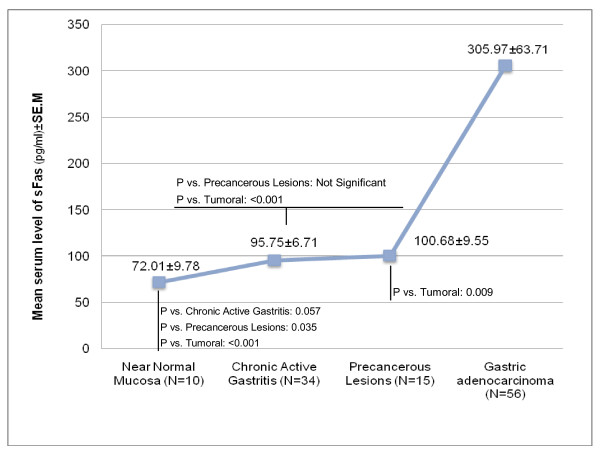
**Mean serum level of sFas (pg/ml) in intestinal-type gastric adenocarcinoma patients and different non-tumoral subgroups**. The graph represents an increasing gradient for the mean serum level of sFas from normal mucosa toward gastric cancer (Kruskal-Wallis test, P < 0.001). Significant difference was observed between serum levels of sFas in each non-tumoral subgroup and cancer group.

An increasing gradient for mean serum level of sFas was observed, from normal mucosa toward gastric cancer (Kruskal-Wallis test, P < 0.001). Analysis showed a significant difference between serum level of sFas in each non-tumoral subgroup and cancer group (Figure 1), (tumoral vs. normal: P < 0.001; tumoral vs. chronic active gastritis: P < 0.001; tumoral vs. precancerous lesions: P = 0.009).

Serum level of sFas/sFasL and their correlation with clinicopathological features of the tumors are represented in Table 4. Although serum level of sFasL was significantly lower in cardiac type of tumor versus non-cardiac type (P = 0.005), serum level of sFas was not significantly associated to tumor location (cardia vs. non-cardia). There was no correlation between serum levels of sFas/sFasL and different histopathologic subtypes of adenocarcinoma (intestinal vs. diffuse), tumor grade of differentiation and stage of tumor. Patients with no lymph node metastasis (N_0_) had significantly higher levels of sFas than those with lymph node involvement (N_1-3_) (P = 0.044).

**Table 4:** Serum levels of sFas/sFasL and their correlations with clinicopathological features of patients with gastric adenocarcinoma

	sFas *(pg/ml)*			sFasL *(pg/ml)*		
		
	N*	Mean ± SE.M^†^	P Value	N*	Mean ± SE.M^†^	P Value
**Tumor invasion**						
*T1 & T2*	5	228.66 ± 50.29	0.45	5	0.46 ± 0.44	0.93
*T3 & T4*	36	227.73 ± 64.32		38	0.09 ± 0.03	
**Lymph node involvement**						
*N0*	14	404.86 ± 150.62	***0.044***	14	0.24 ± 0.16	0.57
*N *>*0*	28	273.00 ± 68.97		30	0.11 ± 0.03	
**Stage**						
*I & II*	22	479.83 ± 151.47	0.2	23	0.22 ± 0.10	0.16
*III & IV*	32	199.70 ± 28.16		34	0.09 ± 0.03	
**Grade**						
*Well Differentiated*	11	392.19 ± 209.08	0.75	11	0.29 ± 0.20	0.92
*Moderatly Differentiated*	15	307.88 ± 136.59		15	0.11 ± 0.05	
*Poorly Differentiated*	4	334.00 ± 232.12		5	0.11 ± 0.08	
**Tumor location**						
*Cardia*	18	242.72 ± 62.78	0.53	18	0.03 ± 0.01	***0.005***
*Non-cardia*	37	358.92 ± 99.61		40	0.21 ± 0.07	

* N: Number of samples

^† ^Mean ± SE.M: Mean Serum Range ± Standard Error of Mean

## Discussion

Gastric cancer is the second leading cause of cancer-related deaths worldwide. It is diagnosed in advanced stages in the majority of cases and no efficient therapeutic modality has been suggested to overcome the problem of treatment resistance yet. Gastric adenocarcinoma has a complex network of molecular alterations along its carcinogenesis pathway. Despite numerous studies focused on this issue, many crucial questions still remain to be clarified. Discovery of these molecular changes can be translated into efficient diagnostic and therapeutic modalities and be employed for targeting the cancer like in some other malignancies[25,26]. Apoptosis-regulating genes play a critical role in carcinogenesis. Fas/FasL system exerts a central role in the apoptosis process and its alterations are noticeable in gastric adenocarcinoma. Although there are some studies indicating that gastric carcinomas express higher levels of FasL and lower levels of Fas to evade the killing effects of host immune system[16-18], there are only few reports addressing their soluble forms.

While Yatsuya *et al *reported significant difference in serum concentration of sFas only between female gastric cancer patients and controls[23], Liang *et al *represented a significantly higher serum level of sFas in all patients with gastric cancer compared to non-tumoral individuals[21], similar to our results. To explain the observation of decrease in Fas and increase in serum level of sFas in gastric cancer[17,21], we hypothesize that translational processing of the *Fas *gene in gastric tumoral cells may be deranged leading to the production of mostly soluble, rather than membranous, Fas. Possibly, mRNA splicing phase alternates toward producing higher levels of sFas with lower molecular weight rather than full-length (membranous) Fas. Thus, we conclude that changes in *Fas *gene expression may be a part of sequential events in the multistep process of gastric cancer development.

To confirm this idea, a group of non-tumoral cases, consisting of various pathologic lesions of non-precancerous and precancerous lesions from different levels of multistep carcinogenesis pathway, were evaluated for serum levels of sFas and sFasL. The findings indicated an increasing gradient in the level of sFas from normal through tumoral epithelium. Analysis showed a significant difference between sFas level in each noncancerous subgroup and cancer group; in line with the findings of Li *et al*[27]. With the application of IHC and western-blot hybridization methods, they reported increasing frequencies of Fas expression in progression from non-cancerous to cancerous mucosa (6.3% in normal mucosa, 60% in atrophic gastritis, 75% in intestinal metaplasia, 100% in grades 2 and 3 dysplasia and gastric adenocarcinoma). They found that soluble Fas (30 KD), but not the membrane type (43 KD), was predominantly expressed in the Fas-positive cases[27]. By quantitatively measuring the increased levels of sFas in serum, we suggest that production of sFas is a crucial event in gastric carcinogenesis. In addition, the significant difference between the serum levels in gastric cancer patients and precancerous group, and also the increases along the carcinogenesis pathway may introduce sFas as a useful, cost-effective, and non-invasive biomarker for early detection of gastric cancer. Further studies with larger sample size are required to establish a precise cut-off point for that purpose. Tamakoshi A *et al *performed a nested case-control study within a large-scale prospective study and suggested that serum sFas has a possibility to detect people at high risk for cancer (regardless of cancer type) prior to diagnosis[15].

We found a significantly higher serum level of sFasL in patients with non-cardiac type of gastric cancer versus those with cardiac type (P = 0.005). To our knowledge, there is no published study addressing the serum levels of sFas/sFasL in cardiac and non-cardiac types of gastric cancer. Our results may confirm the differences in the carcinogenesis pathway and molecular alterations of these subgroups of gastric tumors. Further studies are required to clarify the role of sFas/sFasL in gastric carcinogenesis in cardiac versus non-cardiac tumors.

Some studies reported elevated concentrations of serum sFasL in patients with various types of malignancies and concluded that sFasL may be derived from cancer cells as a result of high expression of *FasL *gene[28-30]. Others have shown lower levels of sFasL in cancer patients compared to controls, suggesting that serum sFasL is possibly consumed by binding to Fas expressed on activated circulating CD8+T lymphocytes[31,32]. In gastric cancer, there are few reports with controversial findings to date. Yoshikawa *et al *showed lower level of sFasL in serum of patients with gastric cancer than normal controls[32], while significantly higher level of sFasL in serum was reported by Ichikura *et al*, only in older male patients (over 50 years old)[20]. In contrast, Tsutsumi *et al *found not statistically significant differences between serum level of sFasL in cancer patients compared to normal individuals[22]. In the studied population, serum level of sFasL was significantly lower in tumoral than non-tumoral group. As the serum level of sFasL could be influenced by both tumor cells production and immune cells consumption of sFasL, the discrepancies in the results may be explained by differences in immune responses of patients. Varieties in clinicopathological features and diversities in socio-demographic characteristics and ethnic background of the studied populations may be another reason for controversial results. Further validation sets focusing on cell expression of FasL, serum level of sFasL and concurrent evaluation of different aspects and impacts of immune response could better elucidate the role of FasL/sFasL in gastric carcinogenesis.

There are few reports about the relation between serum level of soluble Fas and gastric cancer behaviour. Liang *et al *found a direct relation between increasing sFas level with advance in the tumor grade and stage [21]. We observed a lower serum level of sFas in patients with lymph node involvement. When tumor involves lymph nodes, antitumor immunity will be provoked [33] which may result in production of more FasL bearing immune cells and subsequently sFas may be consumed more following binding and neutralizing these Fas Ligands.

Few controversial studies regarding the effects of consuming tobacco on Fas signalling pathways have been published. Some have shown that tobacco increases apoptosis through Fas signalling pathway[34,35], while others reported the anti-apoptotic effects of tobacco on the pathway[36,37]. We found higher levels of sFas in tobacco users of the control group (p = 0.041), which is in favour of apoptotic-inducing role of smoking. However, as most of the cases were not tobacco users (53/62), studies with larger sample size and greater statistical power are required to confirm this finding.

## Conclusions

Fas/FasL system plays a crucial role in gastric carcinogenesis. Assessing the level of sFas in serum, may serve as a biomarker for early diagnosis of gastric cancer. Further studies that investigate both soluble and membranous isoforms of *Fas *gene products may provide valuable information about correlations between serum level and tissue expression of *Fas *gene products, helping in gaining a better understanding of molecular basis of these changes.

## Competing interests

The authors declare that they have no competing interests.

## Authors' contributions

SB-N, HRS, KG, HRR, MJ and JT-A conceived and designed the study, SB-N, KG, carried out the experimental work, SB-N, HH, MJ, OM and MM participated in the classification and characterization of patients, HRS, MTR-M, HH and MM were involved in the recruitment of patients and controls. SB-N, HRS, HRR, MJ, OM and AAA performed the statistical analysis and wrote the manuscript. All authors helped to draft the manuscript and read and approved this final version.

## Pre-publication history

The pre-publication history for this paper can be accessed here:

http://www.biomedcentral.com/1471-2407/10/275/prepub
